# Genotype frequencies for polymorphisms related to chemotherapy-induced nausea and vomiting in a Japanese population

**DOI:** 10.1186/s40780-016-0049-4

**Published:** 2016-07-21

**Authors:** Aya Goto, Haruka Kotani, Masayuki Miyazaki, Kiyofumi Yamada, Kazuhiro Ishikawa, Yasuhiko Shimoyama, Toshimitsu Niwa, Yoshinori Hasegawa, Yukihiro Noda

**Affiliations:** Division of Clinical Sciences and Neuropsychopharmacology, Faculty and Graduate School of Pharmacy, Meijo University, 150 Yagotoyama, Tempaku-ku Nagoya, 468-8503 Japan; Department of Neuropsychopharmacology and Hospital Pharmacy, Graduate School of Medicine, Nagoya University, 65 Tsurumai-cho, Showa-ku Nagoya, 466-8550 Japan; Aichi Health Promotion Foundation, 1-18-4, Shimizu, Kita-ku, Nagoya, 462-0844 Japan; Faculty of Health and Nutrition, Shubun University, 6 Nikko-cho, Ichinomiya, Aichi 491-0938 Japan; Department of Respiratory Medicine, Graduate School of Medicine, Nagoya University, 65 Tsurumai-cho, Showa-ku Nagoya, 466-8550 Japan

**Keywords:** Chemotherapy-induced nausea and vomiting, Gene polymorphism, Japanese population

## Abstract

**Background:**

Genotype frequencies for chemotherapy-induced nausea and vomiting (CINV)-related polymorphisms have not yet been reported for Japanese subjects.

**Methods:**

We analyzed 10 genotype frequencies for following polymorphisms associated with the development of CINV: serotonin 5-HT_3_ receptors (HTR3); neurokinin-1 receptors (Tachykinin-1 receptors, TACR1); dopamine D_2_ receptors (DRD2); and catechol-O-methyltransferase (COMT).

**Results:**

All polymorphisms were successfully genotyped in 200 Japanese subjects and were in Hardy-Weinberg equilibrium. Almost all genotype frequencies were similar to those in the HapMap database or in the previous reports, while frequencies for the Y192H polymorphism in* TACR1 *were different in Japanese subjects from those in a previous report.

**Conclusions:**

The present study revealed genotype frequencies for polymorphisms, which were related to the appearance of CINV in Japanese subjects. Individual therapy based on genotype variations for each race is needed to allow cancer patients to undergo chemotherapy more safely and to understand etiology of CINV.

## Background

Chemotherapy-induced nausea and vomiting (CINV) is a common severe side effect for cancer patients undergoing emetic chemotherapy [1, 2]. CINV is a significant problem because it affects not only the quality of life (QOL) of the patient but also determines the possibility of chemotherapy continuation. Thus, it is extremely important to overcome CINV.

The involvement of 5-hydroxytryptamine (5-HT; serotonin) has been reported as a mechanism of CINV, which is released from enterochromaffin cells in the mucosa of the small intestine adjacent to vagal afferent neurons in response to the stimulation of anti-cancer drugs [3, 4]. The released 5-HT activates serotonin 5-HT_3_ receptors of the medulla via the area postrema and the medial solitary nucleus, ultimately leading to a severe emetic response [5, 6]. Therefore, serotonin 5-HT_3_ receptor antagonists can significantly improve CINV [7]. According to the American Society of Clinical Oncology guidelines, an emetic prophylaxis for high-emetogenic-risk chemotherapy should include a serotonin 5-HT_3_ receptor antagonist, dexamethasone, and aprepitant [8], which, in combination, provides the best antiemetic efficacy [9].

Serotonin 5-HT_3_ receptors are members of the superfamily of Cys-loop ligand-gated ion channels [10]. There are five subunits encoded by different genes in the human genome: serotonin 5-HT_3A_, 5-HT_3B_, 5-HT_3C_, 5-HT_3D_, and 5-HT_3E_ receptors [11–13]. Serotonin 5-HT_3A_ and 5-HT_3B_ receptors are expressed in the hippocampus, spleen, kidney, small intestine, and colon [14]. Serotonin 5-HT_3A_ receptors are mainly involved in the formation of functional receptors [12], and it is the only subunit capable of forming functional homopentameric receptors [15]. The other subunits form functional receptors only when their receptor is co-expressed with serotonin 5-HT_3A_ receptors [16–18].

CINV can also occur via other physiological neurotransmitters, including substance P [19], neurokinin-1 (NK-1) [20, 21], dopamine [21], and catechol-O-methyltransferase (COMT) [22]. Substance P is a member of the neurokinin family of peptides, which includes NK-1. NK-1 receptors (Tachykinin-1 receptors; TACR1) are located in the gut, the area postrema, and the nucleus tractus solitaries [4]. NK-1 exerts its biological effects by acting in the vomiting center in central NK-1 receptors. Thus, NK-1 receptor antagonists have recently been recommended when starting chemotherapy. The COMT enzyme modulates neurotransmission by metabolizing dopamine, which is known to play a role in the development of nausea and vomiting. Dopamine D_2_ receptor blockade in the area postrema and the vomiting center has an antiemetic effect. Thus, it is expected that polymorphisms of the *COMT* would have effects on dopamine-related pathogenesis, treatment, and adverse events [22].

Despite improvements in antiemetic treatment with serotonin 5-HT_3_ receptor antagonists, a considerable number of patients still suffer from CINV. Of patients that received an emetic prophylaxis, 20–38 % and 50–60 % showed delayed nausea and vomiting reactions, respectively [2, 23]. One potential reason for this effect is due to individual genetic differences in the function of their receptors and enzymes. Polymorphisms in their genes could serve as a predictive factor for CINV in patients undergoing moderately emetogenic chemotherapy [24], although there have been no reports confirming this genetic variation in a Japanese cancer patients. In this study, therefore, we analyzed genotype frequencies for polymorphisms of the *HTR3, TACR1*, *DAD2*, and *COMT* genes in Japanese subjects, which are associated with the development of CINV.

## Methods

### Study population

Japanese subjects, who presented at the Preventive Health Service Department of Nagoya University Hospital to have a physical checkup were recruited for this study. The sample of 200 subjects was recruited randomly from unrelated healthy individuals (average age: 49.9 years; range: 25–89 years) of which 125 were male and 75 were female, under institutionally approved internal review board protocols, with informed consent. This study was approved by the ethics committee of Nagoya University Graduate School of Medicine. This study was also performed according to Good Clinical Practice guidelines. The written informed consent documents were obtained from all subjects.

### DNA isolation

Genomic DNA was extracted from peripheral blood using QIAamp® DNA Blood Mini Kit (QIAGEN; Valencia, CA, USA) following the manufacturer’s spin protocol instructions. Purified genomic DNA adjusted to a concentration of 10 ng/μL was stored at −30 °C until analysis.

### Target gene polymorphisms

**Fig. 1:**
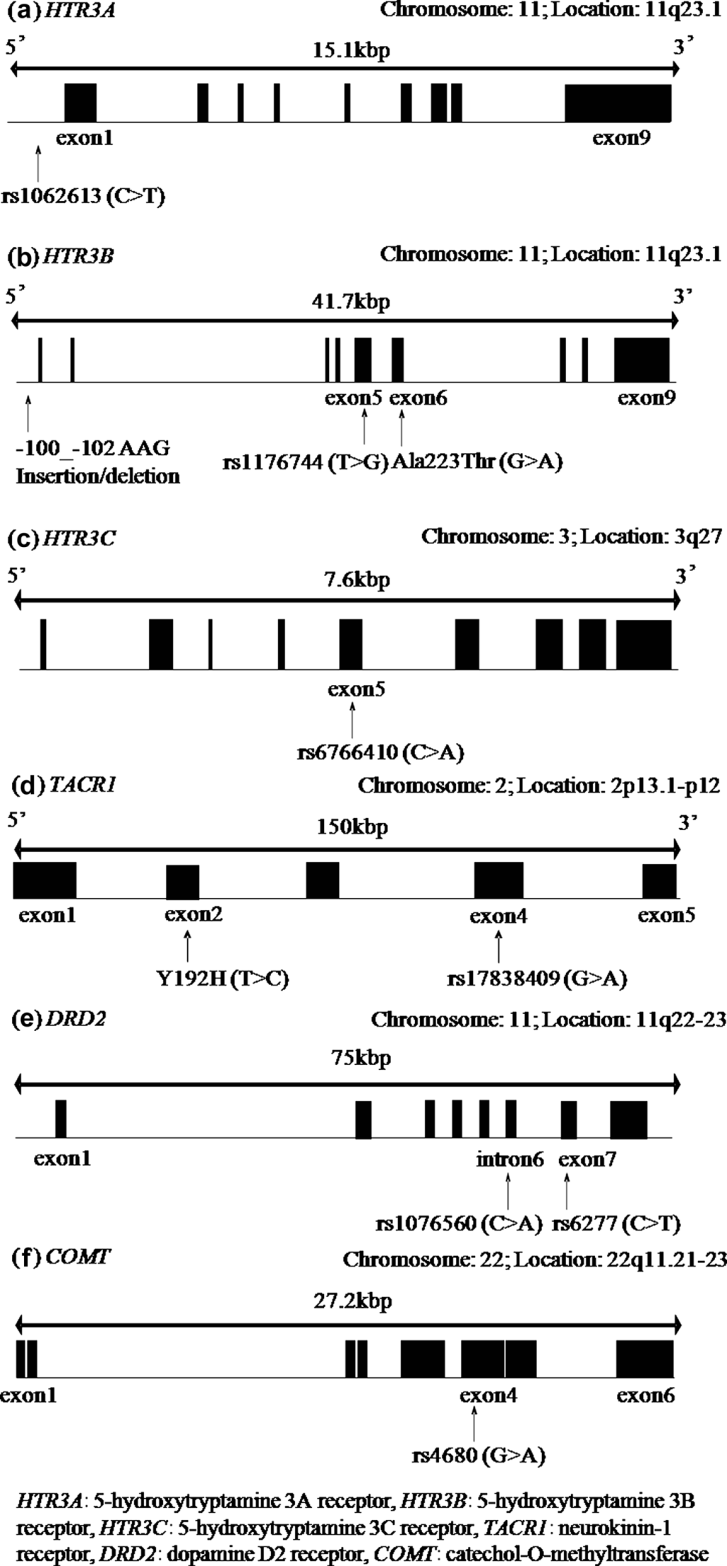
Location of gene polymorphisms. Representative gene structure, location of exons, and polymorphisms in (a) *HTR3A* serotonin 5-HT_3A_ receptors, (b) *HTR3B* serotonin 5-HT_3B_ receptors, (c) *HTR3C* serotonin 5-HT_3C_ receptors, (d) *TACR1* neurokinin-1 receptors, (e) *DRD2* dopamine D_2_ receptors, and (f) *COMT* catechol-O-methyltransferase. For all polymorphisms, the accession numbers of the dbSNP database are indicated

We analyzed the following 10 gene polymorphisms, which are known to be closely related to CINV (Fig. 1).

1. Serotonin 5-HT_3_ receptor genes: *HTR3A* (GenBank accession no. DQ050460) and *HTR3B* (DQ050462) in chromosome 11 (11q23.1), and *HTR3C* (AF459285) in chromosome 3 (3q27). We analyzed the untranslated region of *HTR3A* (rs1062613) [25], the exon regions of *HTR3B* (rs1176744 and Ala223Thr) [25, 26], the promoter region of *HTR3B* (−100_-102AAG deletion) [25], and the exon region of *HTR3C* (rs6766410) [24].
2. NK-1 receptor genes: *TACR1* (AY420417) in chromosome 2 (2p13.1-p12). We analyzed the exon regions of *TACR1* (Y192H and rs17838409).
3. Dopamine D_2_ receptor genes: *DRD2* (AY418851) in chromosome 11 (11q22-23). We analyzed the exon region (rs6277) and the intron region (rs1076560).
4. COMT gene: *COMT* (DQ040245) in chromosome 22. We analyzed the exon region (rs4680) [22].

### Genotyping

Determination of allele variations among subjects was performed using the TaqMan® allelic discrimination assay (TaqMan® 5′-exonuclease allelic discrimination assay; Applied Biosystems; Foster City, CA, USA). The polymerase chain reaction (PCR) mixture contained 1 μL of genomic DNA, fluorescence probes (TaqMan® 20×, 40×, and 80× probes; Applied Biosystems), 2× TaqMan® Universal PCR Master Mix (Applied Biosystems), and distilled water (Wako; Tokyo, Japan) up to a total 10 μL volume in a 96-well microplate. Gene fragments were amplified by PCR using the Applied Biosystems Real-time PCR System. Amplification conditions were as follows: initial denaturation for 10 min at 95 °C, followed by 50 cycles of denaturation at 92 °C for 15 s, and annealing and extension at 58 °C for 1 min.

### Deletion mutation screening

Deletion mutation screening was conducted by direct sequencing analysis. PCRs comprised 1 μL of DNA in the presence of 10× Buffer (TaKaRa; Shiga, Japan), dNTP (2.5 mM dNTPmix; TaKaRa), 20 μM forward/reverse primer (AAG deletion R/F; Rikaken; Nagoya, Japan), and rTagDNA polymerase (TaKaRa). The PCR mixture was amplified with PCR Thermal Cycler Dice® Standard (TaKaRa). The conditions of amplification were 94 °C for 1 min; 40 cycles of 94 °C for 30 s, 55 °C for 30 s, and elongation at 74 °C for 1 min; 1 cycle at 72 °C for 7 min. PCR products were purified from the reaction mixture using distilled water. Cycle sequencing was performed using the Big Dye® Terminator v1, Cycle Sequence Kit (Applied Biosystems) up to 20 μL, and then cleaning step and loaded onto an ABI PRISM^®^ 310 Genetic Analyzer sequencer (Applied Biosystems).

### Statistical analysis

Hardy-Weinberg equilibrium testing was performed using Haploview 4.1 software.

## Results

The genotype and allele frequencies of the polymorphisms are shown in Table 1.

**Table 1:** Genotype distribution

No.	Gene	Localization	SNP	Exchange	N	Genotype frequencies (%)			MAF	HWE
						−/−	+/−	+/+		
1	*HTR3A*	5′ UTR	rs1062613	C > T	200	150 (75.0)	48 (24.0)	2 (1.0)	0.13	0.64
2	*HTR3B*	Promotor	−100_-102 AAG insertion/deletion	delAAG	127	94 (74.0)	27 (21.3)	6 (4.7)	-	-
3		Exon 5	rs1176744	T > G	198	102 (51.5)	83 (41.9)	13 (6.6)	0.28	0.62
4		Exon 6	Ala223Thr	G > A	200	200 (100)	0 (0)	0 (0)	0	-
5	*HTR3C*	Exon 5	rs6766410	A > C	191	79 (41.4)	81 (42.4)	31 (16.2)	0.37	0.80
6	*TACR1*	Exon 2	Y192H	T > C	200	200 (100)	0 (0)	0 (0)	0	-
7		Exon 4	rs17838409	G > A	200	200 (100)	0 (0)	0 (0)	0	-
8	*DRD2*	Exon 7	rs6277	C > T	200	179 (89.5)	21 (10.5)	0 (0)	0.06	0.98
9		Intron 6	rs1076560	C > A	200	76 (38.0)	95 (47.5)	29 (14.5)	0.38	1.00
10	*COMT*	Exon 4	rs4680	G > A	197	88 (44.6)	85 (43.2)	24 (12.2)	0.34	0.87

*HWE* Hardy-Weinberg equilibrium, *MAF* minor allele frequency, *HTR3A* serotonin 5-HT_3A_ receptors, *HTR3B* serotonin 5-HT_3B_ receptors, *HTR3C* serotonin 5-HT_3C_ receptors, *TACR1* tachykinin-1 receptors, *DRD2* dopamine D_2_receptors, *COMT* catechol-O-methyltransferase

### The major alleles

The major alleles were as follows: the C allele for *HTR3A* (rs1062613), the T and G alleles for *HTR3B* (rs1176744 and Ala223Thr), the A allele for *HTR3C* (rs6766410), the T and G alleles for *TACR1* (Y192H and rs17838409), the C allele for *DRD2* (rs6277 and rs1076560), and the G allele for *COMT* (rs4680).

### Genotype frequencies of serotonin 5-HT_3_ receptors

The genotype frequencies of serotonin 5-HT_3_ receptors were as follows. For *HTR3A* (rs1062613), the C/C, C/T, and T/T genotype frequencies were 75.0 % (*n* = 150), 24.0 % (*n* = 48), and 1.0 % (*n* = 2), respectively. For *HTR3B* (rs1176744), the T/T, T/G, and G/G genotype frequencies were 51.5 % (*n* = 102), 41.9 % (*n* = 83), and 6.6 % (*n* = 13), respectively. For *HTR3B* (Ala223Thr), the G/G, G/A, and A/A genotype frequencies were 100 % (*n* = 200), 0 % (*n* = 0), and 0 % (*n* = 0), respectively. For *HTR3C* (rs6766410), the A/A, A/C, and C/C genotype frequencies were 41.4 % (*n* = 79), 42.4 % (*n* = 81), and 16.2 % (*n* = 31), respectively.

On the other hand, for *HTR3B* (−100_-102AAG deletion) (*n* = 127; 73 subjects not detected), the frequencies of insertion/insertion, insertion/deletion, and deletion/deletion mutations were 74.0 % (*n* = 94), 21.3 % (*n* = 27), and 4.7 % (*n* = 6), respectively.

### Genotype frequencies of NK-1 receptors

The genotype frequencies of NK-1 receptors were follows. For *TACR1* (Y192H), the T/T, T/C, and C/C genotype frequencies were 100 % (*n* = 200), 0 % (*n* = 0), and 0 % (*n* = 0), respectively. For *TACR1* (rs1738409), the G/G, G/A, and A/A genotype frequencies were 100 % (*n* = 200), 0 % (*n* = 0), and 0 % (*n* = 0), respectively.

### Genotype frequencies of dopamine D_2_ receptors and COMT

The dopamine-related genotype frequencies were follows. For *DRD2* (rs6277), the C/C, C/T, and T/T genotype frequencies were 89.5 % (*n* = 179), 10.5 % (*n* = 21), and 0 % (*n* = 0), respectively. For *DRD2* (rs1076560), the C/C, C/A, and A/A genotype frequencies were 38.0 % (*n* = 76), 47.5 % (*n* = 95), and 14.5 % (*n* = 29), respectively. For *COMT* (rs4680), the G/G, G/A, and A/A genotype frequencies were 44.6 % (*n* = 88), 43.2 % (*n* = 85), and 12.2 % (*n* = 24), respectively.

### Minor allele frequencies (MAFs)

The MAFs were as follows: 0.13 for rs1062613 (*n* = 200), 0.28 for rs1176744 (*n* = 198; 2 subjects not detected), 0.37 for rs6766410 (*n* = 191; 9 subjects not detected), 0.06 for rs6277 (*n* = 200), 0.38 for rs1076560 (*n* = 200), 0.34 for rs4680 (*n* = 197; 3 subjects not detected), and 0 for the other polymorphisms. There was no deviation from Hardy-Weinberg equilibrium detected (*P* > 0.05).

## Discussions

We investigated genotype frequencies for polymorphisms related to the appearance of CINV in a Japanese population. *HTR3B* (Ala223Thr) and *DRD2* (rs1076560) were particularly first study in Asian. Our results suggest that determining the genotype of these polymorphisms except for *TACR1* and *HTR3B* can help to inform individually based medication for treating or preventing CINV using genomic information for the Japanese cancer patients.

As a general rule, the genotype of a sample used in a genetic analysis must conform to Hardy-Weinberg equilibrium [27]. Sample size (200 subjects) was small to reach conclusive findings, whereas the results could be reliable in Hardy-Weinberg equilibrium, which reflects a population’s actual genetic structure over time with the genetic structure. Our results showed that the genotype frequencies for polymorphisms were similar to those in the HapMap database or reported previously [28], suggesting that they are reliable. Namely, the MAFs of *HTR3A* (rs1062613, *n* = 86, Asian), *HTR3B* (rs1176744, *n* = 90, Asian), *HTR3C* (rs6766410, *n* = 88, Asian), *DRD2* (rs6277, *n* = 82, Asian), *DRD2* (rs1076560, *n* = 98, European), *COMT* (rs4680, *n* = 88, Asian), and *TACR1* (rs17838409, *n* = 226, Asian) were 0.151, 0.30, 0.36, 0.049, 0.12, 0.24, and 0.004, respectively. Although *HTR3B* (Ala223Thr) is not indicated in the HapMap database (http://www.1000genomes.org/), the MAF has been reported to be 0.002 in Caucasians [26].

In a previous study, the *HTR3* polymorphisms were shown to serve as a predictive factor for CINV [28]. Vomiting occurred in 50 % of patients with the C/C genotype of *HTR3C* (rs6766410), compared to only 19 % and 22 % in patients with the A/A and A/C genotypes, respectively [24]. These findings indicated that individual genetic differences affected the response to anti-emetic drugs. Patients with the 100_-102AAG deletion (deletion/deletion) showed vomiting more frequently than those with insertion/insertion and insertion/deletion mutations of this gene [25]. In the present Japanese sample, the −100_-102AAG deletion was not detected in 127 subjects. Although the reasons are unknown, the amount of DNA analyzed might have been too small to detect this deletion. Further investigations are needed to elucidate the role of the −100_-102AAG deletion in relation to CINV in Japanese subjects, including improving method of efficient DNA extraction.

Polymorphisms of the *TACR1* (Y192H and rs17838409) are associated with the binding ability of substance P to NK-1 receptors [29], and have thus far been found only in the African-American population. It is therefore suggested that polymorphisms of the *TACR1* are not clinically relevant for the Japanese population.

Gene polymorphisms have been associated with changing the expression level of gene or protein function. *HTR3A* (rs1062613), which is intronic polymorphism in the 5′ untranslated regions (5′UTRs) of *HTR3A*, affects the expression level of the downstream *HTR3A* [30]. Amino acid substitutions of *HTR3B* (rs1176744 and Ala223Thr) are related to receptor functional disorders [31]. Desensitization of serotonin 5-HT_3_ receptors does not occur in subjects with the *HTR3B* (rs1176744) polymorphism, which changes a tyrosine residue to a serine residue [32]. The T allele in *DRD2* (rs6277) reduces the stabilization of the dopamine D_2_ receptors by changing the folded structure of the mRNA [33]. Thus, reduced dopamine D_2_ receptor (DRD2) binding was found to be associated with the C/C genotype of the rs6277 polymorphism of the *DRD2* gene [34]. Subjects with an A allele in *DRD2* (rs1076560) have a reduced ability to synthesize dopamine D_2_ receptors [35]. A missense variant of *COMT* (rs4680) also leads to an amino acid change. The Val version of *COMT* (G at rs4680) is associated with higher COMT enzyme activity leading to lower levels of dopamine in the brain, while the Met version (A at rs4680) is associated with lower enzyme activity and higher dopamine levels [22].

Determining an individual’s genotype is important to predict the clinical responses to chemotherapy, whereas it is difficult to incorporate the rapidly accumulating genome information for the Japanese population because of genetic differences among races. There are no studies the difference in Japanese and other races for the methods of preventing adverse effects related to CINV. As one of the individual-based medication for adverse effects induced by chemotherapy, UDP-glucuronosyltransferases (*UGT*) 1A1*28 has been suggested to be related to neutropenia induced by irinotecan, a topoisomerase inhibitor used in combination with other chemotherapeutic agents. FDA (Food and Drug Administration) recommends that patients with UGT1A1*28 are treated at the small doses of irinotecan, because the frequency of UGT1A1*28 in Caucasian is higher than that in Asian [36]. We believe that our results help to determine individual-based medications for treating and/or preventing CINV in Japanese cancer patients. Further studies are needed to confirm the relationship between gene polymorphisms and the efficacy of antiemetic therapies on CINV in Japanese cancer patients. These support the idea of establishing individualized supportive therapies (some additional prophylactic antiemetics) for CINV, and contribute to the development of more effective and safer chemotherapies.

## Conclusions

We identified the genotype frequencies for polymorphisms related to the mechanism of appearance of CINV in Japanese subjects. Our study theoretically contributes to increasing the safety of chemotherapy with supportive therapy to prevent CINV and increase the QOL of cancer patients.

## Abbreviations

5-HT, 5-hydroxytryptamine; ABCB1, ATP binding cassette subfamily B member 1; CINV, chemotherapy-induced nausea and vomiting; COMT, catechol-O-methyltransferase; DRD2, dopamine D_2_ receptors; HTR, serotonin 5-HT receptors; MAF, minor allele frequencies; NK-1, neurokinin-1; OPRM1, opioid receptor mu 1; PCR, polymerase chain reaction; QOL, quality of life; TACR1, tachykinin-1 receptors
